# Phase 2a safety and immunogenicity testing of DNA and recombinant modified vaccinia ankara virus vaccines expressing virus-like particles

**DOI:** 10.1186/1742-4690-9-S2-O56

**Published:** 2012-09-13

**Authors:** P Goepfert, M Elizaga, D Montefiori, J Hural, S DeRosa, G Tomaras, K Seaton, A Sato, L Ouedraogo, Y Donastorg, M Cardinali, J Lama, L Baden, M Keefer, J McElrath, S Kalams, H Robinson

**Affiliations:** 1 University of Alabama at Birmignham, Birmingham, AL, USA; 2 Fred Hutchinson Cancer Research Institute, Seattle, WA, USA; 3 Duke University, Durham, NC, USA; 4 NIAID Vaccine Clinical Research Branch, Bethesda, MD, USA; 5 Unidad de Vacunas IDCP-COIN-DIGECITSS, Santo Dominago, Dominican Republic; 6 Asociacion Civil IMPACTA Salud y Educacion, Barranco, Lima, Peru; 7 Brigham and Women's Hospital, Boston, MA, USA; 8 University of Rochester, Rochester, NY, USA; 9 Fred Hutchinson Cancer Research Instituted, Seattle, WA, USA; 10 Vanderbilt University, Nashville, TN, USA; 11 GeoVax Inc., Smyrna, GA, USA

## Background

The Phase 2a HVTN 205 trial was undertaken to further compare full-dose regimens of DNA priming with MVA boosting and MVA priming and boosting.

## Methods

150 vaccinia-naïve participants were inoculated i.m. via needle and syringe with 3 mg of pGA2/JS7 DNA at months 0 and 2, and 1x108 TCID50 of MVA/HIV62B at months 4 and 6 (DDMM regimen). 75 participants received 1x108 TCID50 of MVA/HIV62B at months 0, 2, and 6 (MMM regimen) and 75 received placebo. While the safety data are still blinded, the vaccine regimens appeared safe and well tolerated. Immune studies were performed at 2 weeks following the final vaccination.

## Results

Similar to Phase 1 testing, the DDMM regimen induced higher rates of T cell responses whereas the MMM regimen induced higher rates of antibody responses. CD4 T cell responses were elicited in 65% of the DDMM and 43% of the MMM recipients (p=0.01) whereas CD8 T cells were induced in 22% and 16%, respectively. The majority of T cells were directed against Gag with fewer against Env and only occasional responses to Pol. gp120 IgG antibodies were demonstrated in 45% and 68% of the DDMM and MMM recipients, respectively (p=0.001). gp41 IgG antibodies were seen in over 90% of both groups. The magnitudes of serum IgG responses exceeded the magnitudes of serum IgA responses by >10 fold with higher IgG to IgA responses being present in the MMM group (p=0.03). The antibody avidity index to the gp41 immunodominant epitope, a preclinical correlate of protection against infection demonstrated levels of affinity maturation comparable to preclinical studies. Sporadic weak neutralizing activity against Tier 1 and Tier 2 viruses was seen in both groups and was greater for MVA alone.

## Conclusion

The vaccine safety data and immune responses seen here are supportive of further testing.

